# A Look into the Cell: Honey Storage in Honey Bees, *Apis mellifera*

**DOI:** 10.1371/journal.pone.0161059

**Published:** 2016-08-25

**Authors:** Michael Eyer, Peter Neumann, Vincent Dietemann

**Affiliations:** 1 Agroscope, Swiss Bee Research Centre, Bern, Switzerland; 2 Institute of Bee Health, Vetsuisse Faculty, University of Bern, Bern, Switzerland; 3 Bee Protection Laboratory, Department of Biology, Faculty of Science, Chiang Mai University, Chiang Mai, Thailand; 4 Social Insect Research Group, Zoology and Entomology Department, University of Pretoria, Pretoria, South Africa; University of North Carolina at Greensboro, UNITED STATES

## Abstract

Honey bees, *Apis* species, obtain carbohydrates from nectar and honeydew. These resources are ripened into honey in wax cells that are capped for long-term storage. These stores are used to overcome dearth periods when foraging is not possible. Despite the economic and ecological importance of honey, little is known about the processes of its production by workers. Here, we monitored the usage of storage cells and the ripening process of honey in free-flying *A*. *mellifera* colonies. We provided the colonies with solutions of different sugar concentrations to reflect the natural influx of nectar with varying quality. Since the amount of carbohydrates in a solution affects its density, we used computer tomography to measure the sugar concentration of cell content over time. The data show the occurrence of two cohorts of cells with different provisioning and ripening dynamics. The relocation of the content of many cells before final storage was part of the ripening process, because sugar concentration of the content removed was lower than that of content deposited. The results confirm the mixing of solutions of different concentrations in cells and show that honey is an inhomogeneous matrix. The last stage of ripening occurred when cell capping had already started, indicating a race against water absorption. The storage and ripening processes as well as resource use were context dependent because their dynamics changed with sugar concentration of the food. Our results support hypotheses regarding honey production proposed in earlier studies and provide new insights into the mechanisms involved.

## Introduction

Social insects, incl. honey bees, *Apis* species, display a complex colonial organisation based on division of labour among nestmates, which in particular applies to the acquisition and storage of food [1]. Floral pollen is the main source of protein for the honey bee. Nectar is obtained from flowers and honey-dew is derived from plant-sucking insects [2]. These secretions provide the honey bees with the carbohydrates necessary to maintain their metabolism and conduct specific duties within and outside the hive [3]. Surplus pollen, nectar and honeydew are stored into the cells of the wax combs built by workers. These stores allow honey bees to overcome dearth periods, when foraging is not possible (e.g. during bad weather spells or over winter in the temperate regions). If the processes involved in food collection are well described and understood [4], those leading to the production and storage of honey are poorly understood. This is paradoxical given the importance of this product for colony survival and for beekeeping and trade.

Once brought back to the nest by foragers, carbohydrates are delivered to storer bees, who distribute them to hungry nestmates or process them to produce honey [4]. This ripening process involves physicochemical transformations of nectar during which sucrose is inversed into two simple sugars (dextrose and levulose) by enzymes originating from the hypopharyngeal glands of workers [5,6]. In parallel, water is eliminated to increase sugar concentration [5,6], which is the process we will focus on in this study. The concentration process is driven by active evaporation behaviour by the workers [7–9] and by passive evaporation of cell content under hive conditions [5,10–12]. Ripening dynamics are affected by various parameters such as colony size, amount of available honeycomb cells, movement and humidity of air within the hive, prevalent climatic conditions and botanical origin that determines the ratios of sugar to water content of nectar [5,11,13]. As a consequence of variable interactions between these factors, ripening duration can vary from 1 to 11 days [13,14].

Our knowledge on honey ripening and storing is derived from qualitative descriptions of worker behaviour [7], but measurements of sugar concentration are largely lacking to verify the claims. Moreover, the previous studies designed to investigate these processes prevented further intake of nectar and observations of active ripening [10,11,13], and thus provide only a fragmentary picture of honey production. Concentration measurements also had a limited resolution because they were performed on the pooled contents of several cells [8,13]. More recent studies of carbohydrate storage in honey bee nests used diagnostic radioentomology [15,16], a non-destructive computer tomography based technique allowing measurements of sugar concentration in large numbers of individual cells. With a single snapshot of storage combs, diagnostic radioentomology helped determine whether spatial distribution of storage cells depended on the sugar concentration of their content [17,18].

Here we took advantage of diagnostic radioentomology [16,17,18] to monitor density patterns and measure sugar concentration in individual cells over time. Our aim is to gain a more comprehensive understanding of carbohydrate storage and ripening processes. We worked under more natural conditions compared to previous studies (e.g. [8,10,11,13]) by using free-flying colonies in which cell filling and ripening were both performed by the workers. To be able to control food provisioning of the test colonies as well as to mimic natural conditions when foraging occurs on plants secreting nectar of different qualities [11,19–21], solutions with different sugar concentrations were provided. Based on the observation of cell use, food consumption and density measures of cell content, we determined how sugar concentration and filling status of storage cells changed throughout the ripening process to form honey. We deduced the cell filling and ripening behaviour of workers indirectly from the pattern of content density observed in single cells. We also aimed at testing the hypothesis that cell content relocation is an integral part of ripening process [5,11]. Relocation can only be linked to ripening if the concentration of content removed is lower than that of content deposited subsequently. Another process we lack information on, is the final stages of honey production, i.e. the capping of the cell over the ripe content [1]. As nectar is concentrated during ripening, it becomes hygroscopic [5,22] and can therefore absorb water from the hive atmosphere, which can lead to its fermentation [23,24]. Parallel concentration and capping processes of individual storage cells therefore appear essential for efficient ripening by reducing the absorption surface. To gather evidence towards such a potential race again honey dilution during the capping process, we here test the hypothesis that ripening is still ongoing as the workers seal the cells with wax. For this, we measured sugar concentration during and after the sealing of the honey cells. Our results will help broaden our understanding of a unique hoarding behaviour, which constitutes a central adaptation of honey bees to overcome dearth periods, especially in the temperate regions.

## Material and Methods

### Study design

From June to August 2012, three free-flying local honey bee colonies of mixed European origin were used for the study (~4,000–5,000 workers, one mated and ovipositing queen) in Bern, Switzerland (Latitude: 46.967223; Longitude: 7.397433). Since *A*. *mellifera* is not a protected species, no specific permission was required to perform the study. In order to control the carbohydrate supply of each colony and to correlate it to storage patterns, the study was conducted during a nectar dearth and sugar water was provided in feeding trays within the hives.

The test colonies were housed in small sized Miniplus (R) hives [30 × 30 × 34 cm, with 5 frames], thereby enabling to focus the X-ray beam on a limited surface area for an enhanced scanning resolution. The combs containing brood and pollen (with occasional traces of nectar and honey, < 2% of cells) were left in the hives, but the peripheral honey combs were replaced with newly drawn empty combs (hereafter designated as the “test combs”) to promote and monitor carbohydrate storage. No smoke was used during colony inspections to limit nectar take up by workers, which would interfere with naturally occurring storage processes.

Each hive was equipped with a Miniplus (R) feeding tray in which two containers [Ø = 13 cm, height = 6 cm] were placed. In order to study the storage of food covering most of the range of sugar concentration encountered during foraging (15%–65% [19,20]), 30% and 70% sugar-water solutions were provided. Given that no knowledge is currently available to determine which conditions favour honey cell filling and capping, the feeding regime was adjusted according to the observations on storage progress during the study. From day one to three, we fed daily 200 ml volumes of 30% and 70% sugar solution to each colony. From day 4 onwards, we increased the quantity of both solutions given to ensure satiation and therefore promote storing and thus the need for ripening. From day five to six, colonies were only provided with 30% sugar solution in a further attempt to promote ripening behaviour. Subsequently, from days 7 to 10, the colonies were only provided with 70% sucrose solution, to promote sealing of honey cells. The amount of food consumed and stored was determined daily by weighing the containers placed in the feeder and weighing the test combs after brushing the bees away.

To confirm that the colonies were hungry and thus willing to use the food provided during the study and to identify any potential bias from other nectar sources available to the free-flying colonies, we assessed the nectar flow by measuring the crop sugar concentrations of 5 returning workers daily for each colony. For this, we compressed their abdomens dorsoventrally, leading to regurgitation of crop contents [9]. The droplet obtained was placed in a refractometer and its sugar concentration measured [21].

### Diagnostic radioentomology

Scan images of the test combs were obtained with a Philips Brilliance CT 16-slice scanner (Philips Healthcare, 5680 DA Best, The Netherlands) using routine protocols [15,16,18] at 18°-20°C. CT scans were performed between 13:00 and 15:00 on the first, second, fifth, eight, and twelfth day after the introduction of the empty wax-combs and of the feeders. A regression of sugar concentrations on density of the solutions was used to convert density Hounsfield units in % sugar concentration [18]. To describe the patterns observed, we used the following terminology for cell architecture: cell foundation refers to the vertical mid rib of the comb, representing the bottom of a wax cell. The term cell opening represents the open end of the cell.

#### Visual analyses

To allow for the visualisation of cell content density patterns, we analysed the CT-images with BeeView 3D software (Disect Systems Ltd; Suffolk, UK). The following settings for the windowing feature were used: Level: 75 and Width: 505 with colour feature *black on white*. For each test comb, a plane was chosen that crossed a maximum number of cells of which the diameter was completely filled with sucrose solution. In order to avoid air and wax inclusions, the selected plane was at least 3 scanning frames (a plane of 3 dimensional pixels perpendicular to the long axis of the cell) away from cell foundation and 3 frames away from cell opening.

#### Density analyses

We recorded density in Hounsfield Units (HU) with the ellipse tool of *e*FilmLite version 1.5.0.0 [18]. In brief, an ellipse was drawn over the content of each cell without including any wax material of the cell walls. Preliminary measures showed within cells variation of density. To take into account this variation, we averaged HUs measurements of two ellipses per cell: at 3 scanning frames distance to the bottom of the cell and three frames away from the surface of cell content, where no wax or air was present.

### Observations

#### Patterns of content density within individual cells

Since worker behaviour was not directly observed, storing and ripening behaviour was instead derived from the density patterns of the content of individual cells filled from a quarter to a half. These patterns were described in terms of homogeneity, presence of speckles, dark or bright appearance. Screening of cells of the three combs scanned on day 1 was performed until no new visual pattern of density could be identified. To determine how these patterns changed over time, we selected five cells of each pattern category and also screened them on day 2.

#### Filling and ripening dynamics at the individual cell level

Preliminary observations showed that cells that started to be filled after the test comb was placed in the hive were rarely capped at the end of the study period. It was thus not possible to follow the complete filling and ripening processes in a single cohort of cells, i.e. those that showed some content at day 1. We thus monitored two cohorts, one with cells that contained solution on the first day, thereafter referred to as “early provisioned cells” and a second with cells that were capped at the end of the study, thereafter referred to as “eventually capped cells”. We selected 10 early provisioned cells and 10 eventually capped cells on each test comb (N = 3). Content concentration in each of the 20 cells was measured at days 1, 2, 5, 8 and 12. E-Film was used for these measurements, as described in the section “diagnostics radioentomology”. On these dates, the filling status of the cells was also estimated as the number of scanning frames for which the content filled the whole cell diameter.

To investigate in detail the last stage of cell content ripening, we compared the density of the content of completely capped cells (N = 29) with that of cells that workers had started to seal with wax, but that still showed holes of various sizes in their capping (thereafter referred to as “partially capped cells”, N = 30).

#### Ripening dynamics at the comb level

**Fig 1:**
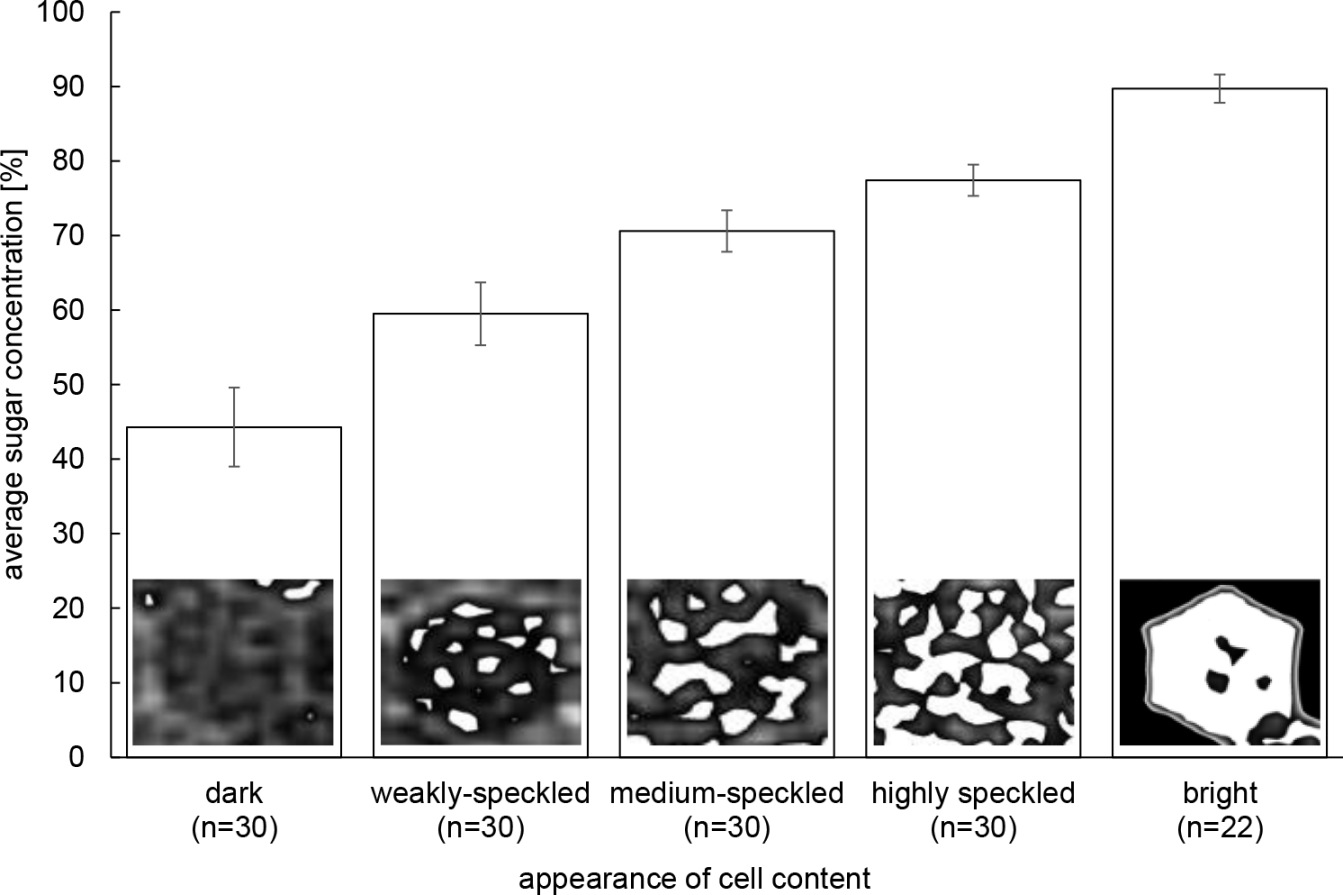
Visual categories of cell content density patterns. Average **±** S.D. sugar concentrations (in %) for 22–30 cells per category is shown. The sugar concentrations in the various visual categories of cell content density were normally distributed (Anderson-Darling: *P* ≥ 0.07) and thus compared with an ANOVA. All categories of sugar concentrations were significantly different from each other (post-hoc Scheffé multiple comparison tests: *P* < 0.001).

In order to measure changes in sugar concentration over time with a higher sample size than what is possible with the direct measurements of cell content HUs, we visually categorized density patterns in 117–877 cells per comb (total N = 7,225) using Disect. To ensure that the accuracy of the visual pattern identification was sufficient to describe the phenomenon studied, we validated it by measuring with E-Film the HU of a subsample of N = 22–30 cells (total N = 142) of the different categories determined visually (see Fig 1). There was an increasing and non-overlapping sugar concentration for cell displaying homogeneously dark content, dark content with a few bright speckles, dark content with medium or high numbers of bright speckles and entirely bright cells (Fig 1). The different patterns identified visually therefore represented a valid index of distinct sugar concentration ranges. We quantified the number of cell showing these pattern categories on one side of each of our 3 test combs for each scan (at days 1, 2, 5, 8 and 12).

### Statistical analyses

Parametric linear mixed models applied to the repeated measures of individual cells showed strong deviations of the residuals from normality (Shapiro-Wilk, Anderson-Darling, and Lilliefors tests, *P* < 0.05), therefore all analyses performed to study the dynamics of cell filling and content concentration between early provisioned and eventually capped cells were performed applying robust rank-based methods [25]. For this, we used an ANOVA-like statistic based on the package nparLD [26] of R [27]. This test’s statistics distribution approximates the F-distribution [26] and is referred to as ‘statistic’ in the results section. Similarly, for the following analyses, whenever residuals were not normally distributed, non-parametric tests were used.

Ripening dynamics were further investigated by comparing percentages of cells with different attributes (filling level increasing vs. not increasing, content concentration increasing vs. not increasing, slow vs. fast filling) between early provisioned and eventually capped cells with Pearson Chi-square tests. With the same aim, we used the Wilcoxon-Mann-Whitney test to compare the changes in counts of low (dark and weakly-speckled cells) and high density (medium-, highly-speckled and bright cells) cells over time as well as to compare the properties of the two cell types studied between consecutive days or periods. Since for these comparisons single days were used for two comparisons (one with the previous and one with the following day), we applied a Bonferroni correction. This test was also used to investigate the resource use of the colonies by comparing the mass of food stored between consecutive days as well as by comparing the mass of solution removed from the feeders with that stored in the test combs. To assess the effect of cell relocation on ripening, we also used the Wilcoxon-Mann-Whitney test and compared the sugar concentration and level of filling of cells that were subsequently emptied with that of cells suddenly filled. To increase our understanding of the last stages of honey production, a two-sample t-test compared the sugar concentration in partly and completely capped cells. The program SYSTAT 13 was used for these analyses.

Ripening dynamics were also investigated at the comb level by comparing density pattern proportions (considered as ordered categories: dark, little, medium, high, bright) between colonies and over time with the R function polr() (generalized linear models for proportional odds logistic regression [28]; R Core Team 2016). Comparisons of proportions of each pattern between two consecutive days in unpaired data structures were done with two-sample tests for equality of proportions (prop.test) of package stats; R Core Team 2016). Poisson models were applied for pairwise comparisons of total counts of cells filled between consecutive days using the log-link in glm) (R Core Team 2016).

## Results

### Natural availability of food during the study period

It took up to 15 min to gather the 5 workers with sufficient crop load to determine natural availability of nectar. The nectar gathered by foragers was of low concentration (N = 148, median = 31% sucrose, 1^st^ quartile: 28.0; 3^rd^: 66.8).

### Patterns of content density within individual carbohydrate storage cells

**Fig 2:**
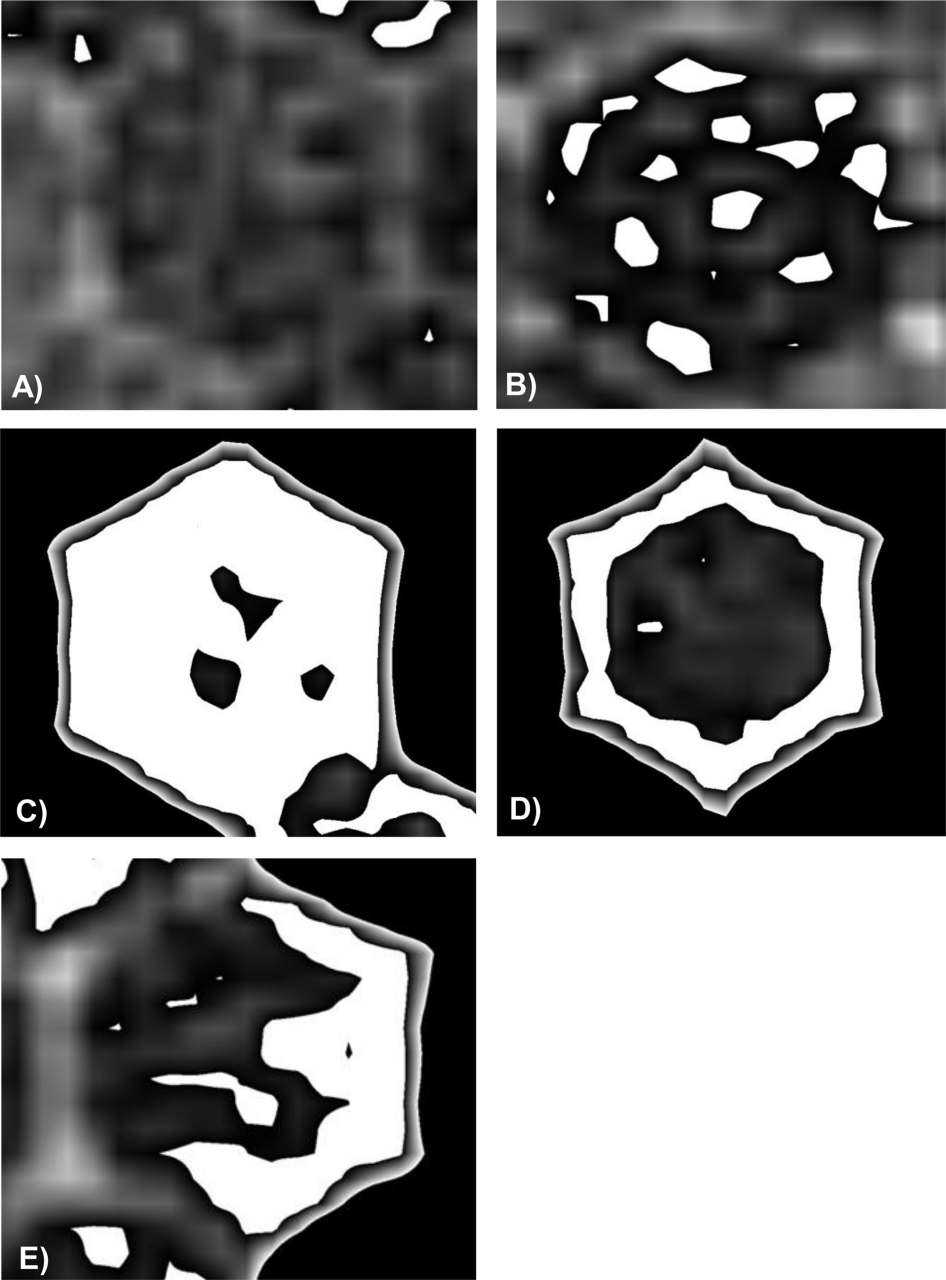
Patterns of content appearance observed in individual cells at day 1. A) dark cell content of low density, B) cell content contains a variable number of high density speckles of varying sizes, C) most of the content of bright cells is of high density, D) the high density content coats all or E) some of the walls.

One day after the colony was given access to the feeders, distinct density patterns emerged within the cells. One pattern consisted in cells displaying entirely dark (low density) content (Fig 2A), while another consisted in bright (high density) speckles spread within the cells (Fig 2B). The number and size of speckles could vary until the cell content was mostly bright (Fig 2C). A further pattern was represented by high-density content at the periphery of the cells, along all (Fig 2D) or some cell walls (Fig 2E). Three quarters of the cells that showed the latter three high-density patterns (Fig 2C–2E) were neighbouring empty cells (S1 Fig).

Visual inspection of individual cells (N = 5 per pattern category, Fig 2A–2D) over time revealed several content density dynamics. Dark cells generally changed to a speckled appearance, but were occasionally emptied. Cells showing homogeneously bright content at day 1 remained bright at day 2, changed to speckled appearance, or were occasionally emptied. Initially speckled cells showed an increase in speckle numbers and size at day 2, but on few occasions a decrease was observed. The highly concentrated material along the walls of certain cells (Fig 2D) could disappear and result in a speckled appearance or thicken to the point where the whole cell showed a bright content. From day 5 onwards and until day 12, we observed only speckled cells that kept this appearance, became entirely bright or were emptied.

**Fig 3:**
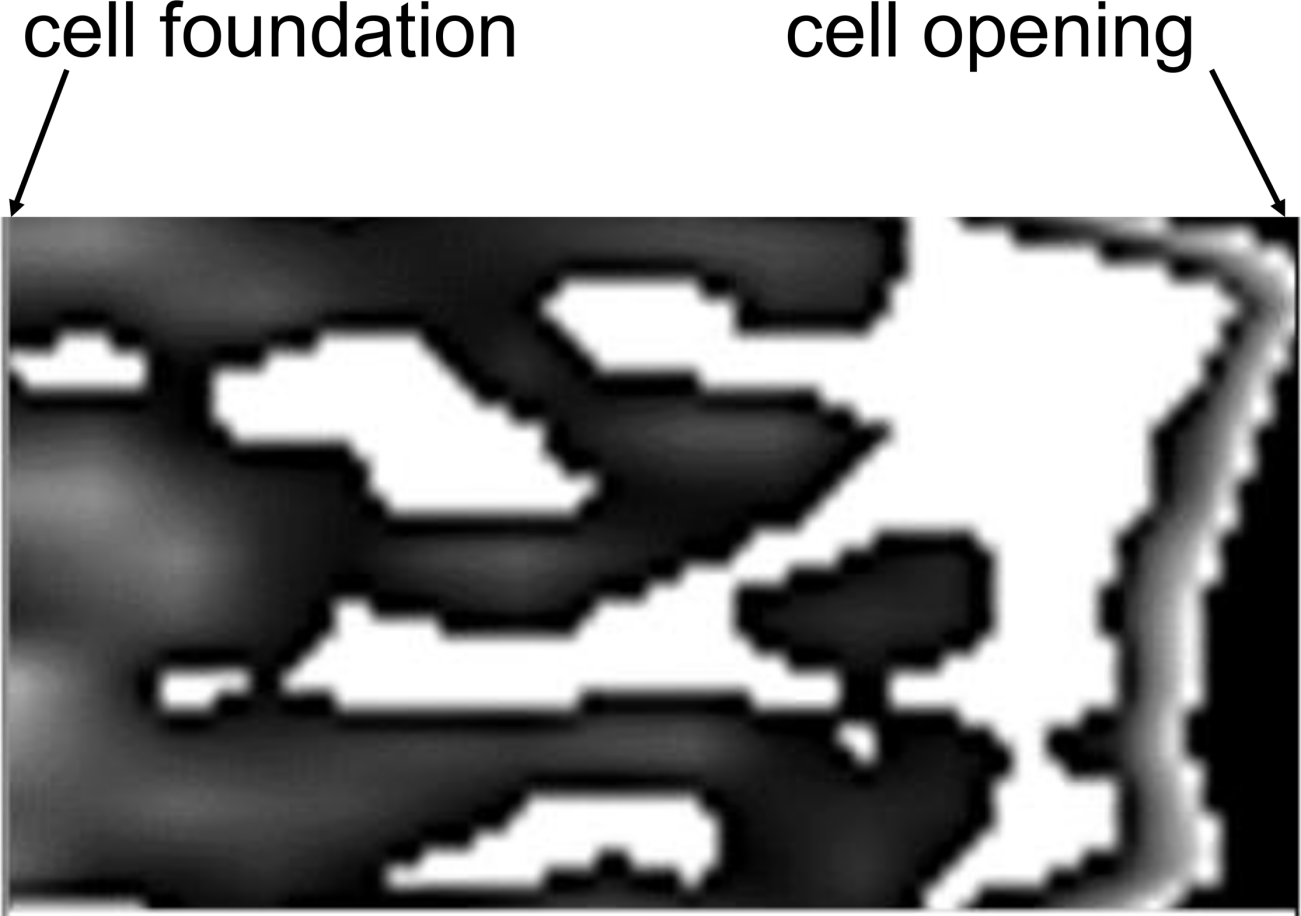
Sagittal view of a cell with content density increasing towards the cell opening (on the right side).

**Fig 4:**
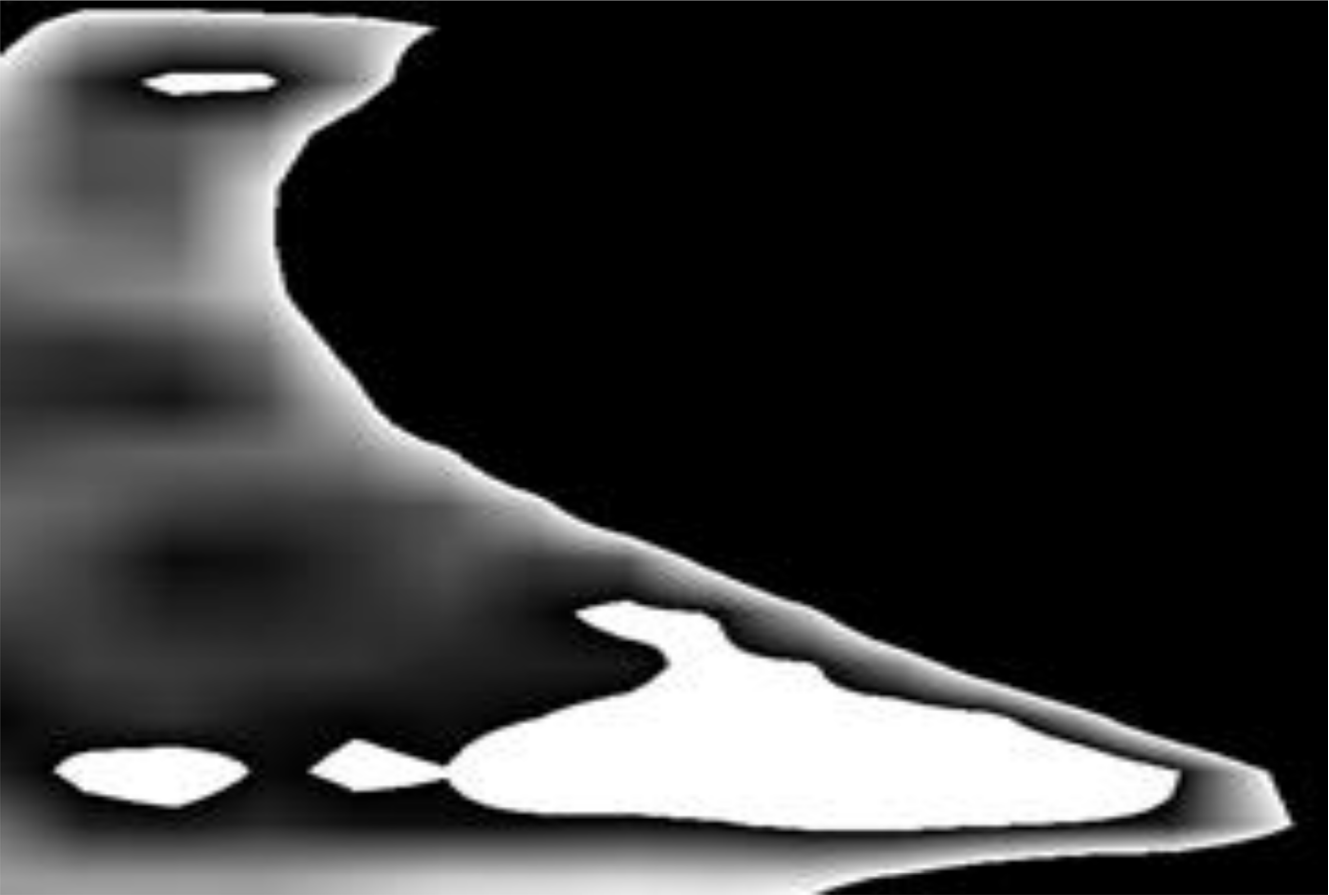
Sagittal view of partially filled cell. A ‘tongue’ of high-density content is visible at the cell opening.

In sagittal section, irrespectively of observation day, most of cells showed variables patterns of content appearance. For instance, a variation of speckle density could occur along the long axis of the cell. Approximately a fifth of the cells showed increasing speckle density from the *cell foundation* to the *cell opening* (Fig 3). Sugar concentration of 73.6 ± 2.1% measured near the foundation and 91.4 ± 1.4% near the opening confirmed this observation. We also observed ‘tongues’ of dense solution towards the opening of partly filled cells (Fig 4).

### Filling and ripening dynamics at the individual cell level

**Fig 5:**
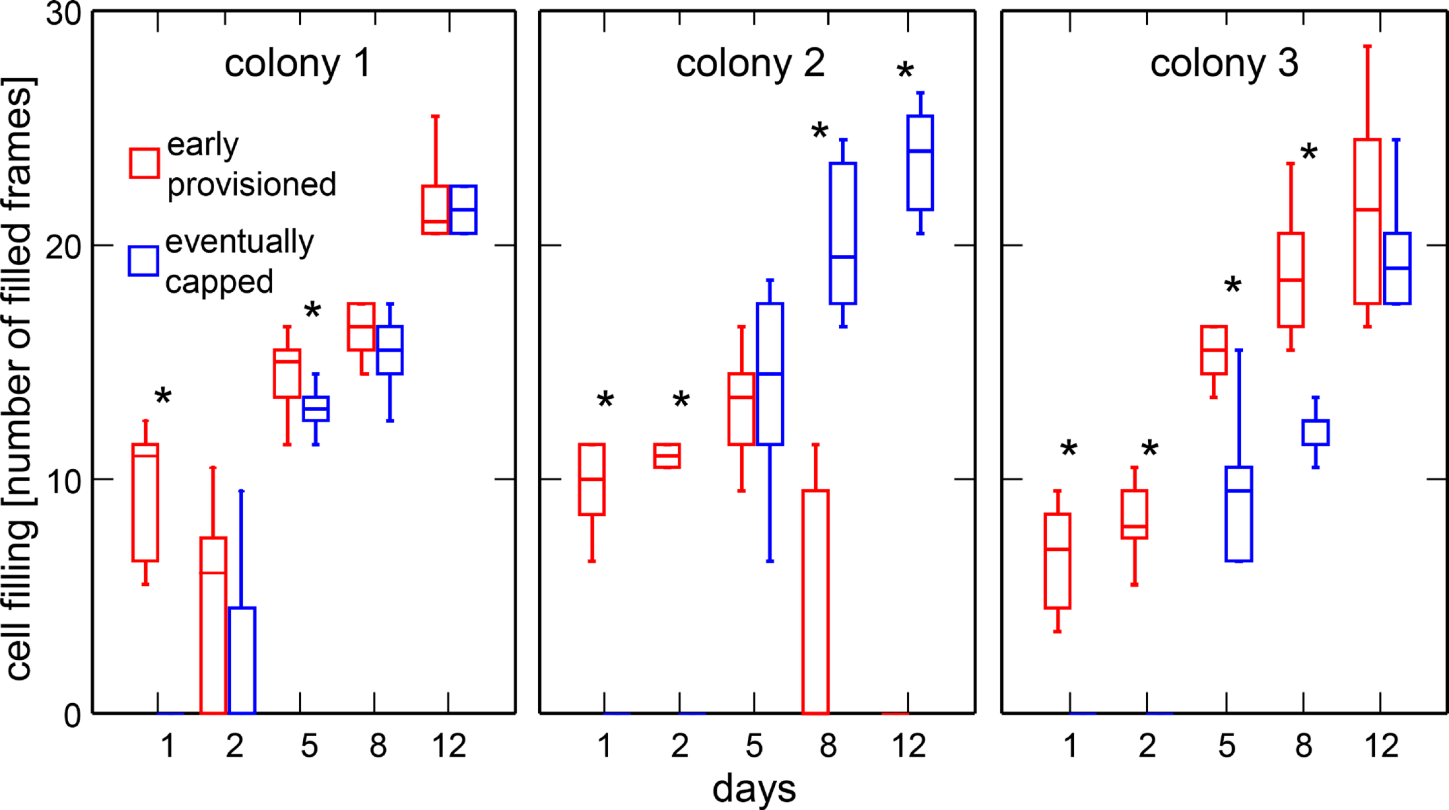
Comparison of the filling levels of cells provisioned early and eventually capped. Medians, first and third quartiles as well as range are shown. Sample size is 10 cells per box. For clarity, outliers identified by Systat are not shown. Significant differences between cell types are indicated with * (Wilcoxon-Mann-Whitney test, *P* < 0.025).

We measured an average increase in concentration of 10.6% between day 1 and 2 in 31 cells showing content on day 2. In 49 cells showing content at day 12, the daily average increase (between days 5 and 12) in concentration was 1.8% (U = 7.73; df = 1; *P* = 0.005). Only 10% (3 out of 30) of the sample of cells that were provisioned by workers on the first day were capped after 12 days. Conversely, few (2 out of 30, 6.6%) of the second sample of cells that were capped at the end of this period showed content on the first day. Content deposition in most cells eventually capped (25 out of 30, 83%) started after day 2 (Fig 5). We thus analysed these cohorts of cells (early provisioned and eventually capped) separately.

The robust rank-based method showed that the amount of solution filled in the cells between scans is significant different between the early provisioned and eventually capped cells (statistic = 6.05; df = 1.0; *P* = 0.014). Colonies showed significant differences in the amount filled per scan (statistic = 12.84; df = 2.0; *P* < 0.001; Fig 5). The interactions between colony and cell type as well as that between colony, cell type and days were significant (statistic = 57.87; df = 2.0; *P* < 0.001; statistic = 34.57; df = 4.8; *P* < 0.001, respectively). Filling of cells followed a similar pattern in two colonies (#1 and 3) where a continuous increase occurred both in early provisioned and eventually capped cells. The increase was significantly higher in early provisioned cells compared to eventually capped cells for six scans out of 10 (S1 Table). In the third colony (#2), filling of early provisioned cells decreased significantly between days 5 and 8 (statistic = 30.16; df = 1; *P* < 0.001, S2 Table) and was significantly higher in eventually capped cells at days 8 and 12 (Fig 5, S1 Table).

**Fig 6:**
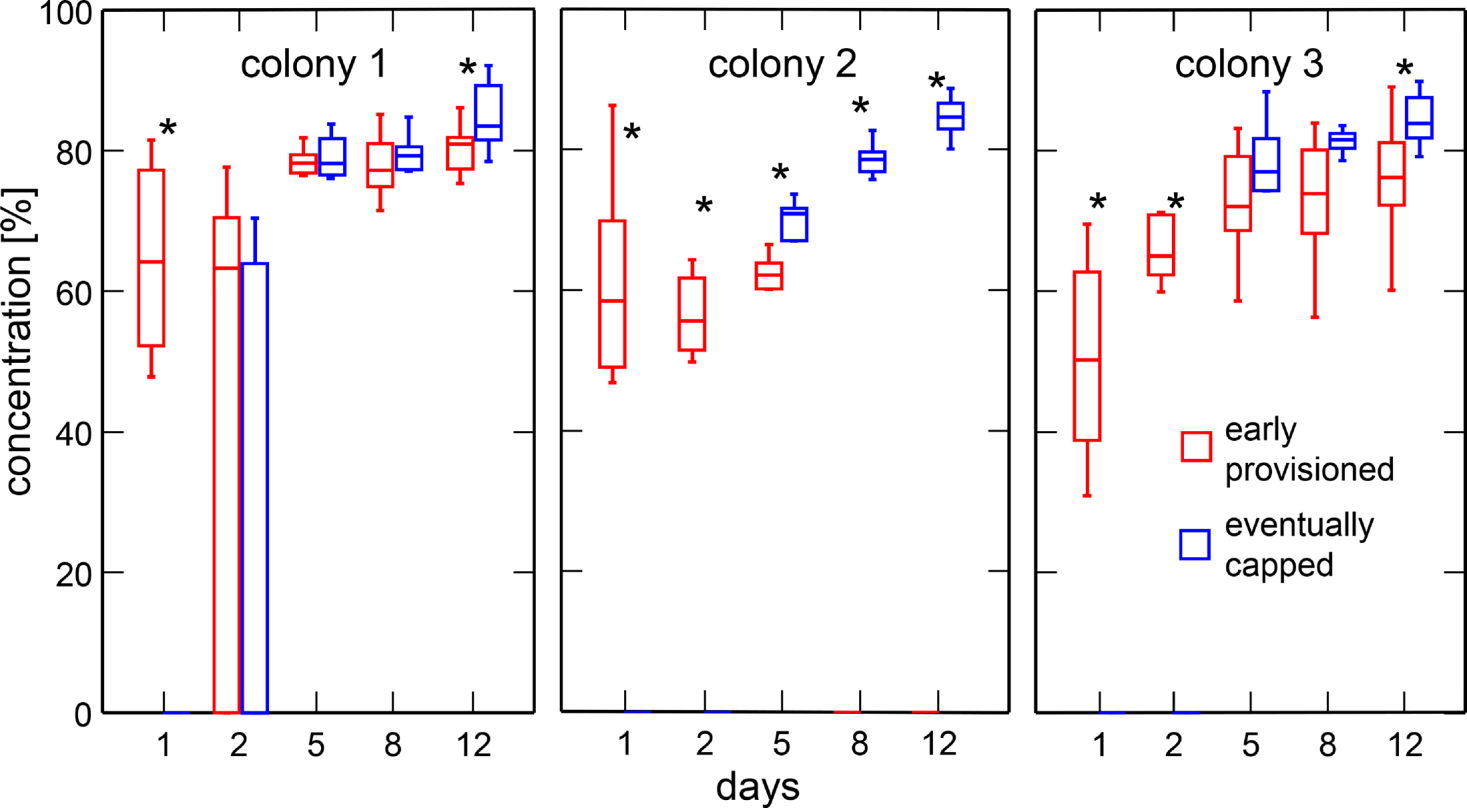
Comparison of sugar concentrations between cells provisioned early and eventually capped. Medians, first and third quartiles as well as range are shown. Sample size is 10 cells per box. For clarity, outliers identified by Systat are not shown. Significant differences between cell types are indicated with * (Wilcoxon-Mann-Whitney test, *P* < 0.025).

For concentration of cell content, the robust rank-based method used shows that values at each scan are different between early provisioned and eventually capped cells (statistic = 4.98; df = 1.0; *P* = 0.025). Colonies showed significant differences in the content concentration of cells over the study period (statistic = 33.09; df = 1.7; *P* < 0.001). The interactions between colony and cell type as well as that between colony, cell type and days were significant (statistic = 11.98; df = 1.7; *P* < 0.001; statistic = 5.70; df = 5.70; *P* < 0.001, respectively). On the first day of feeding, the median concentration of cell content in early provisioned cells was of 57.7% (N = 30; range: 31–86; Fig 6). Once most eventually capped cells showed content (from day 5 onwards), their sugar concentration was higher than that of early provisioned cells but only significantly so for five scans out of 10 (Fig 6; S1 Table). At day 12, the sugar concentration was always significantly higher in the cells eventually capped (Fig 6; S1 Table). In these capped cells, sugar concentration reached a median of 84.8% (N = 30; range: 79–92; Fig 6).

Filling and sugar concentration did not continuously increase in all of the 60 cells measured over the 12 days and differences in dynamics were measured between the early provisioned and capped cells. The percentage of cells showing increasing filling level was significantly higher for cells eventually capped (29 out of 30, 96.7%) than for cells provisioned early (19 out of 30, 63.3%) (Chi-square test: χ^2^ = 10.4, df = 1, *P* = 0.001). The percentage of cells in which sugar concentration increased over time was higher for cells eventually capped with 80.0% (24 out of 30), compared to 56.7% (17 out of 30) of the early provisioned cells. This difference was marginally significant (χ^2^ = 3.77, df = 1, *P* = 0.05).

It took 4–7 days for most cells eventually capped (25 out of 30, 83.3%) and 11–12 days for the remaining 16.6% (5 out of 30 cells) to reach the maximum level of filling observed at the end of the study period. In contrast, for most cells provisioned early (15 out of 20, 75%; 10 cells were empty at the end of the study period), it took 11–12 days to reach an equivalent filling level. The remaining 25% of these cells (5 out of 20) were already full after 4 to 7 days. The proportion of cells filled rapidly was significantly higher in the cells that were eventually capped (χ^2^ = 17.01, df = 1, *P* < 0.001).

Over the entire study, fifteen out of the 60 cells sampled (both provisioned early and eventually capped) showed content at a given scan time, but were found empty in the next scan (e.g. S2 Fig). At the last time point they still showed content, their median concentration was 63.8% (1^st^ quartile: 61.0; 3^rd^: 66.8; range: 33–81%), with a median filling level of 10.5 frames (1^st^ quartile: 6.5; 3^rd^: 14; range 4–16). In contrast, 30 empty cells were provisioned between two scans. These cells showed a median content concentration of 76.8% (1^st^ quartile: 73.8; 3rd: 79.6; range: 55–88%) and a median filling status of 12.5 frames (1^st^ quartile: 10.8; 3rd: 14.5; range 6–18). The concentration of the newly provisioned cells was significantly higher than that of the content of cells before they were emptied (Wilcoxon-Mann-Whitney test: U = 81.0; df = 1; *P* < 0.001). In contrast, their filling status was not significantly different (U = 155; df = 1; *P* = 0.09).

Sugar concentration in partly capped cells (81.1 ± 3.2%) was significantly lower than in the cells for which the capping process was complete (84.8 ± 3.3%; two sample t-test: t = -4.1; df = 56.9; *P* < 0.001).

### Ripening dynamics at the comb level and resource use

**Fig 7:**
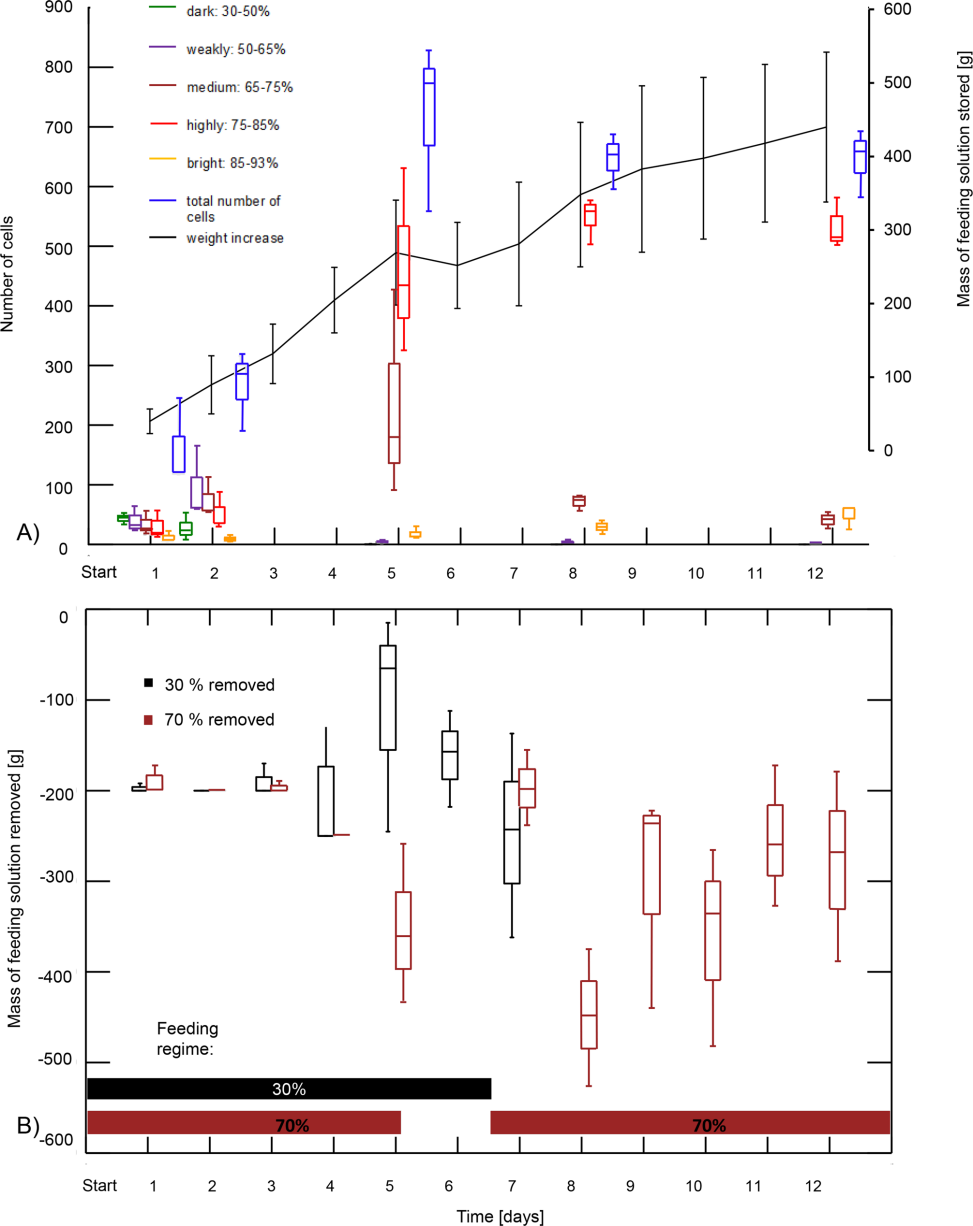
A) **Number of cells filled with content of different sugar concentrations** (see Fig 1) **and mass of stored solution over time** (average ± S.D.). Box plots represent median, first and third quartiles as well as range. B) **Mass of feeding solutions (30% and 70% sugar concentration) removed from the feeders over time**. Box plots represent median, first and third quartiles as well as range. The horizontal bars in the lower part of the graph illustrate the feeding regime.

The three colonies differed significantly in the proportions of cells observed in each category of density pattern over time (proportional odds logistic regression: χ^2^ = 338.56; df = 2; *P* < 0.001). The interaction between colony and time (scan day) was significant (χ^2^ = 134.84; df = 2; *P* < 0.001). Statistical comparisons between the proportions of density categories observed from one scan day to the next are given in the supplementary material (S3 Table). The number of dark cells with a sugar concentration of 44.3 ± 5.3% (average ± S.D) significantly decreased until day 5 (Fig 7A, data of the three colonies graphically pooled; S3 Table for comparisons for each colony), despite the presence of low concentration food until this day (Fig 7B). Between day 1 and 2, the number of weakly-speckled cells with a sugar concentration of 59.5 ± 4.2% increased, but only significantly so in one colony (S3 Table), and significantly decreased from day 2 to 5 (Fig 7A, S3 Table). The number of medium-speckled cells with a sugar concentration of 70.6 ± 2.8% increased until day 5. This increase was significant between day 1 and 2 in one colony, and in one colony between day 2 and 5, while it decreased significantly in the two others during the latter period (S3 Table). In the following days, the number of these cells significantly decreased in all cases but one, where the decrease was not significant between days 8 and 12 in colony 3 (*P* = 0.085; S3 Table). The number of highly-speckled cells with a sugar concentration of 77.4 ± 2.1% increased significantly in all colonies from day 2 to 8 to stabilise thereafter (Fig 7A, S3 Table). The increase in number of cells in the high density range (corresponding to medium-, highly-speckled and bright cells) between days 2 and 5 was greater than the corresponding decrease in the number of cells with lower density patterns (dark and weakly-speckled cells). The difference in cell numbers between days 2 and 5 corresponds to a ratio of 5.5 versus 0.05 for high and low density cells, respectively, but this difference was not significant (U = 29; *P* = 0.08). The amount of bright cells with a sugar concentration of 89.7 ± 1.9% was generally low (Fig 7A). The proportions between consecutive days were not significantly different in nine cases out of 12 but a significant increase occurred in the three remaining cases (Fig 7A, S3 Table). In all colonies, the total number of cells used for storage increased until day 5 (statistically significant in all cases except in colony 1 between days 1 and 2), then decreased between days 5 and 8 (significantly in colonies 2 and 3) and stabilised after this day in colonies 1 and 3, while it decreased significantly in colony 2 (Fig 7A, S4 Table). The weight gain of the test comb stagnated between days 5 and 6 when colonies had only access to the 30% feeding solution (Fig 7A and 7B), but increased until and after this period. However, this increase was not significant between consecutive days (S5 Table).

The amount of solution stored represented 10% (range: 8 to 12%, Fig 7B) of the solution removed from the feeders and was thus significantly lower than the latter’s amount (U = 37; df = 1; *P* < 0.001). From day 8 onwards, bees were only fed with 70% sucrose solution, which they removed from feeders in large quantity (70.6 ± 14.2% of the 1,500g provided; range: 756–1,384g).

## Discussion

The various patterns of cell content density observed in storage combs varied over time. This density could increase, decrease or remain stable in individual cells, but at the comb level, a general increase in filling level, content concentration and number of storage cells was observed. Cell content appeared inhomogeneous throughout the ripening process and until after cell capping. Changes over time in filling level and speed as well as in sugar concentration differed in two types of cells: those provisioned early and those that were capped at the end of the observation period. Content relocation was at the origin of these two types of cells. Sugar concentration in cells that would later be emptied was lower than that of cells filled after relocation. In the last stage of honey production, significant differences in sugar concentration occurred between cells for which capping was ongoing and cells for which it was completed. Significantly higher values were measured in fully capped cells. Our data also showed that only a tenth of the feeding solutions provided to the test colonies was stored in the combs. The latter increased in weight mostly when 70% sugar solution was provided and not when only 30% sugar solution was given. Low concentration solutions seemed to be preferentially consumed, whereas high concentration solutions were stored. Despite significant differences in dynamics or filling, ripening and relocation between colonies, we could derive the behavior of storer bees from our observations and thus better understand how honey is produced.

### Resource use by honey bees: consumption versus storage

Overall foraging activity was low during the study period. It was thus unlikely that large amount of natural food source was more concentrated and more attractive than that provided. As a consequence, most of the food stored by the colonies was likely to be that provided. Weight of stored solution was 10% than that of the solutions removed from the feeder, indicating that the greater proportion of solutions provided was consumed by the colonies during the dearth period when the study took place. Such ratio may vary over the year due to environmental factors influencing nectar production [21,29] as well as colony hunger status. Honey storage should thus be studied over the entire foraging season under different environmental contexts to provide a better understanding of resource use versus storage by honeybee colonies. As indicated by significant interactions between the parameters measured in both cell types and the factor colony, there may be yet unresolved intercolonial differences in ripening dynamics. Since we have not measured the humidity within the test hives, this factor could have varied between colonies and influenced the concentration process [12].

The observations of density patterns revealed that both high and low concentrated feeding solutions were provisioned into cells in the beginning of the study. Later, no more low density solution was found in the combs, which could indicate that this solution was concentrated before being deposited [11]. Alternatively, but not mutually exclusive, the provided 30% feeding solution was more readily consumed by the bees rather than stored. This is supported by the absence of mass increase of the test comb when only 30% solution was provided on days 5 and 6 (Fig 7). Preferably consuming sugar solutions of low concentration is also advantageous since the optimal sugar concentration for consumption is below that of honey. Indeed, workers dilute honey before consuming it [30]. However, it is currently unknown which concentration is required for consumption. Preferably, storing solution of high concentration also appears adaptive since honey ripening effort is reduced when starting from a higher concentration. The concentration threshold determining whether forage will be consumed or stored could also vary depending on the amount of food already stored in the colony [4], on environmental factors (foraging season versus winter or abundant nectar flow versus dearth) and on physiological or genetic factors of individual workers [31].

### Deriving nectar processing behavior of workers from density patterns of cell content

One day after feeding, several density patterns could be observed within cells that had been provisioned. Two patterns consisted of homogenous cell content: dark cells provisioned with low density content (Fig 2A) or bright cells with high density content (Fig 2C). Both patterns reflected bulk filling of content of similar sugar concentration in individual cells. The occurrence of homogeneously dark cells corresponds to the storage of low density feeding solution that has not been subjected to concentration process. This is in line with the observations that during a major nectar flow, bees do not always process nectar, but instead store it immediately to cope with the high number of foragers returning and needing off-loading [4,7].

Cells with inhomogeneous (speckled) content were commonly observed in both vertical and sagittal sections (Figs 2 and 3). The number and size of speckles varied considerably among individual cells (ranging from weakly to highly speckled cells), showing different stages of content concentration. Alternatively, this inhomogeneous appearance could have resulted from the deposition in the same individual cells of 30% and 70% sugar solutions. This possibility is supported by earlier results showing that workers mix solutions of different sugar concentrations during the storing process [18]. Additional evidence for mixing of cell content between different cells was provided in the present study by the occurrence of cells in which content density was not increasing (fluctuating density in 21% and decreasing density in 3.5% of cells, respectively) over time. Such variations can only occur after addition of a less concentrated solution into a cell. Other cells with inhomogeneous content were characterised by high-density content along their walls (Fig 2D and 2E). This pattern may result from the ‘wall painting’ behaviour [7] by which workers release nectar on the cell ceiling with sideways head movements (much as in painting behaviour). Subsequently, the nectar could run down the cell walls in a thin layer, generating the high-density wall coating. Park [7] also described that if cells were already partly filled with nectar, workers added more nectar by dipping their mouthparts into the content already present. Accordingly, if nectar of lower density were added to a ‘painted’ cell, the observed ring pattern might result.

Cells with inhomogeneous content show provisioning with different sugar qualities and indicate that thorough homogenisation does not occur during cell provisioning. The majority of capped cells, which are no longer processed by workers, also showed a content of inhomogeneous density, indicating that final stored product (*i*.*e*. honey) is not a homogenous carbohydrate solution. This has not been described before and is comparable to the heterogeneous ‘bee bread’ stored in the combs. Bee bread is composed of layers of pollen originating in the deposition of pollen pellets of different botanical origins in the same cell [32].

### Ripening processes

Concentrating processes can rapidly increase cell content by at least 16% (86% sugar concentration was measured 24h after feeding, whereas a maximum of 70% sugar concentration was provided). Since passive evaporation of naturally stored nectar under hive conditions increased concentration from only 2 to 8% [8], the occurrence of an active concentration process is likely to explain the increase observed. During ‘tongue lashing’ behaviour [7,33], workers concentrate droplets of regurgitated nectar with movements of their mouthparts that can result in a rise of 10 to 25% sugar concentration of the nectar collected within a few hours [8]. However, since our study design uncoupled behaviour from the resulting density patterns observed, we could not unambiguously determine which patterns resulted from active or passive mechanism or from a combination of both. A similar limitation prevented determining whether the thin highly concentrated layer of feeding solution formed by tension forces at the cell-opening was the result of passive ripening due to a high surface to volume ratio, or of active ripening of the first cell content available for processing by approaching workers. Extending this argument to the ‘painted cells’, the dense ring of content could be due to the deposition of nectar of low concentration, which while flowing down the cells walls exposes a high surface area to passive evaporation or to deposition of actively ripened nectar on the walls. Such ringed cells (as well as cells with homogeneous bright content), were often located along the edges of the storage areas or adjacent to empty cells. It is thus possible that concentration of their content was promoted by metabolic heat produced by workers entering the neighboring empty cells to rest [1]. Whether this phenomenon is a consequence of workers entering cells specifically to promote ripening in an analogous mechanism to brood heating [34] is not known. Our combs were freshly built and therefore devoid of larval silk that could have absorbed water [35] at the contact zone between cell wall and content. It is therefore unlikely that water loss through the hydrophobic wax walls could have created these patterns.

Under colony conditions, the passive concentration process of experimentally filled storage comb is faster for smaller sugar solution volumes, displaying a larger surface area [10,11]. In these studies, the increase in concentration within 24 hours was higher in cells that were a quarter full (~65%), compared to those filled up to three quarters (~30%, [10,11]). Our results support the hypothesis that bees exploit this physical property: in the first two days of our investigation performed under more natural conditions than previous studies, the filling status of cells remained at a low levels, with an increase of concentration of 10.6% per day. In contrast, concentration increased of only 1.8% per day after day 5, when cells were more than half full. However, in absence of behavioral observations, we cannot exclude that active ripening contributed to the initial increase of sugar concentration.

### Relocation of cell content as part of the ripening process

The total number of provisioned cells on the combs increased until day 5 and decreased until day 8 to stabilise until the end of the study. The parallel constant increase in weight of the comb indicates that cell content was gathered and pooled into fewer cells. The individual cell observations reflect this relocation since half of the early provisioned cells were emptied over the study period. This is in line with earlier observations that bees tend to store the fresh nectar in small amounts in many cells spread widely over the comb as long as there is enough space available, before the nectar is collected again and put into the cells in a more clustered pattern [5]. Whether cell content is concentrated during the relocation process was so far unclear since no measure of sugar concentration of cell content before and after relocation had been performed [5]. Our data indicate that the cell content is indeed actively concentrated by workers before being relocated into a different cell. An alternative explanation is that the cell content displaced is mixed with more concentrated solution obtained from the 70% feeder before being relocated. Diagnostic radioentomology did not allow us to distinguish between these possibilities. In addition, one might argue that cell content of lower concentration was consumed instead of concentrated and redeposited. Since only 10% of the solution removed from the feeder was stored and 90% was consumed, this scenario seems unlikely. Most of the workers nutritional need should have been covered by direct consumption from the feeder. Our observations are therefore likely to reflect only ripening and storage processes, rather than consumption from stored reserves.

In one colony (#2), relocation was associated with brood production. Workers emptied storage cells in the centre of the test comb to clear space for brood rearing. This contributed to the higher rate of relocation observed in this colony, but was accompanied by an increase in concentration of the remaining cells, as in the other colonies. This highlights the need to consider the overall colony condition to identify the rules determining honey ripening, including the spatial constraints due to comb use for other purposes than nectar storage. Based on CT scanning at four day intervals, relocations appear to be frequent. Since relocation events most likely also occurred between scans, our estimate is conservative. Future measurements at closer intervals are required to trace relocation events at a higher temporal resolution.

### Partitioning of comb in processing and storage cells

Early provisioned cells were frequently emptied during the study and their content relocated in other cells that were thus suddenly filled and finally capped. Comparison of filling levels and content concentration between early provisioned- and capped cells revealed significant differences. A significant interaction with the factor colony indicated different dynamics between each test unit. However, general observations could be derived from the observations. The cell filling level was in general significantly higher in cells provisioned early until day 5, whereas content concentration tended to be higher in the eventually capped cells after this day (Figs 5 and 6). After day 5, the proportion of cells showing an increase in filling status in the eventually capped cells was also significantly higher, as well as the speed with which they were filled. Despite similar cell filling levels observed at day 8 and 12 in both groups of cells in colonies 1 and 3, early provisioned cells were not about to be capped since the concentration of their content did not reach that of honey.

Our observations suggest that the content of two cohorts of cells varies in both quantity and concentration over time. Those cells that were not capped until the end of the study functioned as transient storage cells during the ripening process. In contrast, cells that were filled later and significantly faster with highly concentrated content were eventually capped and constituted the final honey store. Such a spatial segregation between processing and storage cells via content transfer from the former to the latter may foster the honey ripening process and be economic due to size limitations of the available comb area.

### Final stages of ripening

To this date, there was no documented threshold value of sugar concentration at which a honey cell is capped by workers. Our measures showed a sugar concentration of 84.8 ± 3.5% (range: 79–92%) for cells capped within the last 24h. These values correspond to the higher range given in the literature for ripe honey (75–86% [36]). Under our study conditions, capping of storage cells was first observed at day 12, while other studies reported a faster completion of ripening (1 to 11 days [13,14]). These differences could be explained by factors internal and external to the colony. For example, the dearth period and the resulting need for direct consumption coupled with the access to food within the nest may have prolonged the process by affecting the perception of storage needs by the workers. It is also possible that methodological differences explain the slower ripening. In contrast to other studies, we placed feeders inside the hives and they could have acted as a source of water increasing humidity. Since the rate of evaporation from the cells varies inversely to the relative humidity in the hive’s atmosphere [13], ripening process could have slowed down. Our results show higher sugar concentrations in capped vs. partly capped cells. This is not expected if cell capping is initiated only once their content is ripe. In this case, concentration in partly capped cells should be similar to that of fully capped cells. Our results therefore indicate that the ripening process is still ongoing when capping has already started and hence suggest the possibility of a race against honey dilution.

## Conclusions

Variation in ripening dynamics within and between individual cells might reflect the outcome of storer bees’ behavior to work around the spatial and biophysical constraints to nectar ripening. The reorganization of stores to concentrate nectar into honey and the coordination with other colony functions, such as brood rearing [37], is achieved via cell content relocation. This process led to the overall increase of filling level in a high number of storage cells, of which the content concentration eventually reached that of honey. Ripening dynamics showed intercolonial variations that might be explained by internal factors (e.g. genetics, variations in humidity within the hives [12]). In conclusion, our data provide evidence for the occurrence of both passive and active mechanisms involved in honey production and confirm previous hypotheses based on behavioural observations [5,10,11]. Given the high resolution and non-destructive property of the method used, we were also able to describe previously unknown phenomena of nectar processing and honey production at the individual cell and comb levels (e.g. within cell heterogeneity of content and their dynamics over time). A combination of diagnostic radioentomology and detailed behavioral observations under a range of natural conditions will help to further advance our understanding of the ripening and storing of honey according to the colony’s nutritional and environmental contexts and contribute to improving beekeeping management.

## Supporting Information

S1 Fig Test comb appearance in colony 2. Scans were performed at A) day 1, B) day 2, C) day 5, D) day 8, E) day 12 after feeding. Cell density patterns observed on each day are depicted by icons on the right side of each picture. Note 1) the increasing density and number of filled cells; 2) the changing shape of the area of nectar containing cells due to the relocation of cell content after workers cleared cells for brood rearing (empty central area in D and E); such changes (1 and 2) also occurred in the other two colonies but with a lower frequency; 3) the dense areas of cell content neighbouring empty cells.
(TIF)

S2 Fig Sugar concentration (left y-axis) and filling level (right y-axis) over time in ten individual cells per colony. Each row corresponds to a colony and shows a representative subsample of filling and ripening dynamics. The first five cells of each line represent early provisioned cells that contained solutions already at day 1 (some were relocated at a later stage); the following 5 cells represent eventually capped cells.
(TIF)

S1 Table Results of Wilcoxon test comparing the filling and content concentration of early provisioned and eventually capped cells at each scan day. Significant *P—*values (< 0.025) after Bonferroni correction are indicated with *.
(DOCX)

S2 Table Comparison of cell filling level and content concentration between consecutive days in early provisioned and eventually capped cells. Significant *P—*values (< 0.05) from the robust-ranked method (nparLD) are indicated with *.
(DOCX)

S3 Table Results from the two-sample tests for equality of proportions between consecutive days. The test was performed for each pattern category and for the three colonies separately. Decrease or increase of proportions between the two days are indicated with < and >, respectively. Significant *P—*values (< 0.05) are indicated with *.
(DOCX)

S4 Table Results of the Poisson models (log-link in glm(), R) applied for pairwise comparisons of total number of cells filled between consecutive days in each colony. Decreasing and increasing values are indicated with < and >, respectively. Significant *P*-values (< 0.05) are indicated with *.
(DOCX)

S5 Table Wilcoxon-Mann-Whitney test comparing the amount of solution stored in the three colonies between consecutive days. (DOCX)
