# Supplementary figures and images for: A Look into the Cell: Honey Storage in Honey Bees, Apis mellifera
Source: PLoS One. 2016 Aug 25;11(8):e0161059. doi: 10.1371/journal.pone.0161059 (PMC4999132; doi:10.1371/journal.pone.0161059)

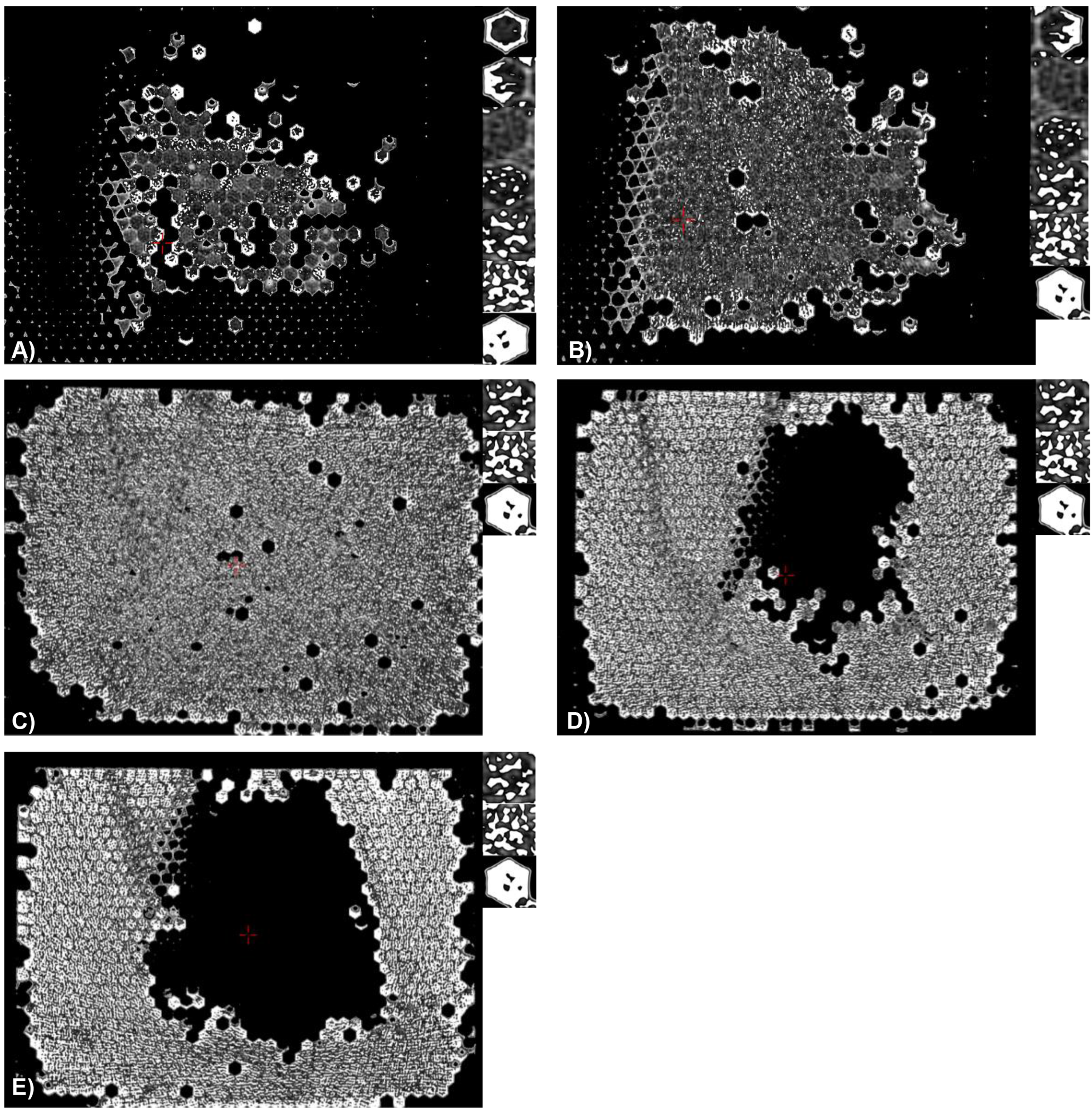

Supplement: S1 Fig — Scans were performed at A) day 1, B) day 2, C) day 5, D) day 8, E) day 12 after feeding. Cell density patterns observed on each day are depicted by icons on the right side of each picture. Note 1) the increasing density and number of filled cells; 2) the changing shape of the area of nectar containing cells due to the relocation of cell content after workers cleared cells for brood rearing (empty central area in D and E); such changes (1 and 2) also occurred in the other two colonies but with a lower frequency; 3) the dense areas of cell content neighbouring empty cells. (TIF) [file pone.0161059.s001.tif]

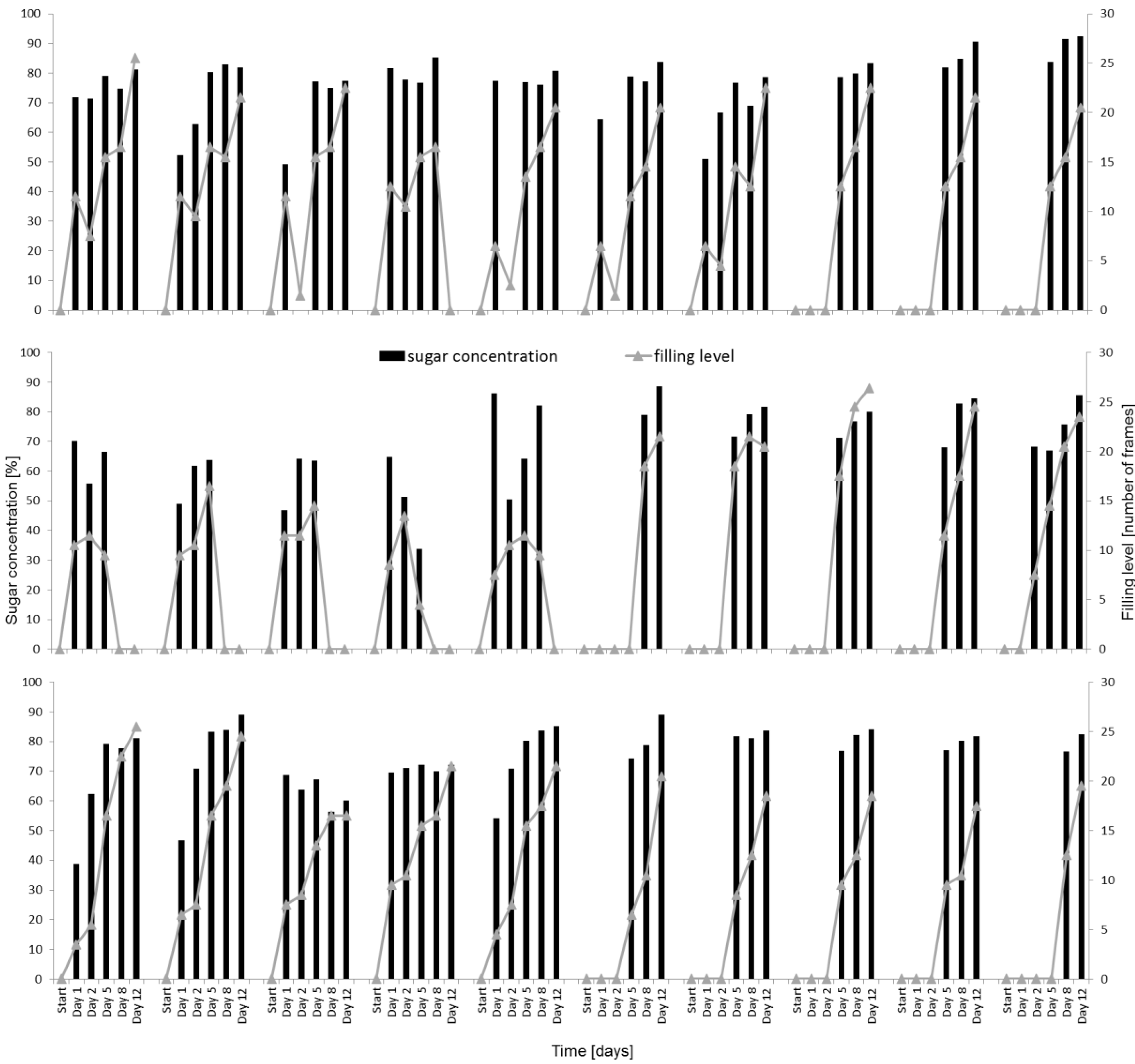

Supplement: S2 Fig — Each row corresponds to a colony and shows a representative subsample of filling and ripening dynamics. The first five cells of each line represent early provisioned cells that contained solutions already at day 1 (some were relocated at a later stage); the following 5 cells represent eventually capped cells. (TIF) [file pone.0161059.s002.tif]
