# Supplementary material for: A Look into the Cell: Honey Storage in Honey Bees, Apis mellifera
Source: PLoS One. 2016 Aug 25;11(8):e0161059. doi: 10.1371/journal.pone.0161059 (PMC4999132; doi:10.1371/journal.pone.0161059)
Supplement: S1 Table — Significant P—values (< 0.025) after Bonferroni correction are indicated with *. (DOCX) [file pone.0161059.s003.docx]

|  |  | **Cell filling** | | **Content concentration** | |
| --- | --- | --- | --- | --- | --- |
| **Colony** | **Day** | **W** | ***P* - value** | **W** | ***P* - value** |
| 1 | 1 | 95.0 | <0.001* | 91.0 | 0.001* |
| 1 | 2 | 66.5 | 0.17 | 67.5 | 0.15 |
| 1 | 5 | 81.0 | 0.02* | 55.0 | 0.71 |
| 1 | 8 | 66.0 | 0.21 | 36.5 | 0.31 |
| 1 | 12 | 44.0 | 0.63 | 19.5 | 0.02* |
| 2 | 1 | 100.0 | <0.001* | 100.0 | <0.001* |
| 2 | 2 | 99.5 | <0.001* | 90.0 | 0.002* |
| 2 | 5 | 35.5 | 0.26 | 18.0 | 0.02* |
| 2 | 8 | 0.0 | <0.001* | 11.0 | 0.002* |
| 2 | 12 | 0.0 | <0.001* | 0.0 | <0.001* |
| 3 | 1 | 100.0 | <0.001* | 100.0 | <0.001* |
| 3 | 2 | 99.5 | <0.001* | 98.0 | <0.001* |
| 3 | 5 | 94.5 | 0.001* | 39.0 | 0.41 |
| 3 | 8 | 85.5 | 0.01* | 22.0 | 0.04 |
| 3 | 12 | 68.5 | 0.16 | 17.0 | 0.01* |
