# Supplementary material for: A Look into the Cell: Honey Storage in Honey Bees, Apis mellifera
Source: PLoS One. 2016 Aug 25;11(8):e0161059. doi: 10.1371/journal.pone.0161059 (PMC4999132; doi:10.1371/journal.pone.0161059)
Supplement: S2 Table — Significant P—values (< 0.05) from the robust-ranked method (nparLD) are indicated with *. (DOCX) [file pone.0161059.s004.docx]

|  |  |  |  | **Cell filling** | | | | | | **Concentration of cell content** | | | | | |
| --- | --- | --- | --- | --- | --- | --- | --- | --- | --- | --- | --- | --- | --- | --- | --- |
| **Cell type** | | | | **Early provisioned** | | | **Eventually capped** | | | **Early provisioned** | | | **Eventually capped** | | |
| **Colony** | **Day** | | | **Statistic** | **df** | ***P* - value** | **Statistic** | **Df** | ***P* - value** | **Statistic** | **Df** | ***P* - value** | **Statistic** | **df** | ***P* - value** |
| 1 | 1 and 2 | | | 13.71 | 1 | <0.001* | 0.98 | 1 | 0.32 | 1.39 | 1 | 0.24 | 1.18 | 1 | 0.28 |
| 1 | 2 and 5 | | | 121.62 | 1 | <0.001* | 191.49 | 1 | <0.001* | 31.15 | 1 | <0.001* | 45.46 | 1 | <0.001* |
| 1 | 5 and 8 | | | 11.35 | 1 | 0.001* | 16.92 | 1 | <0.001* | 0.38 | 1 | 0.53 | 1.07 | 1 | 0.30 |
| 1 | 8 and 12 | | | 19.83 | 1 | <0.001* | 89.55 | 1 | <0.001* | 1.13 | 1 | 0.29 | 20.25 | 1 | <0.001* |
| 2 | 1 and 2 | | | 1.75 | 1 | 0.19 | 1.00 | 1 | 0.32 | 0.15 | 1 | 0.70 | 1.00 | 1 | 0.32 |
| 2 | 2 and 5 | | | 2.67 | 1 | 0.10 | 34.39 | 1 | <0.001* | 2.44 | 1 | 0.12 | 21.90 | 1 | <0.001* |
| 2 | 5 and 8 | | | 30.16 | 1 | <0.001* | 68.98 | 1 | <0.001* | 6.13 | 1 | 0.01* | 11.56 | 1 | 0.001* |
| 2 | 8 and 12 | | | 2.68 | 1 | 0.10 | 12.53 | 1 | <0.001* | 2.24 | 1 | 0.13 | 47.02 | 1 | <0.001* |
| 3 | 1 and 2 | | | 16.00 | 1 | <0.001* | 1.00 | 1 | 0.32 | 10.68 | 1 | 0.001* | 1.00 | 1 | 0.32 |
| 3 | 2 and 5 | | | 34.09 | 1 | <0.001* | 30.24 | 1 | <0.001* | 13.19 | 1 | <0.001* | 27.99 | 1 | <0.001* |
| 3 | 5 and 8 | | | 24.77 | 1 | <0.001* | 43.77 | 1 | <0.001* | 0.16 | 1 | 0.69 | 8.11 | 1 | 0.004* |
| 3 | 8 and 12 | | | 30.27 | 1 | <0.001* | 34.40 | 1 | <0.001* | 12.96 | 1 | <0.001* | 4.91 | 1 | 0.03* |
