# Supplementary material for: A Look into the Cell: Honey Storage in Honey Bees, Apis mellifera
Source: PLoS One. 2016 Aug 25;11(8):e0161059. doi: 10.1371/journal.pone.0161059 (PMC4999132; doi:10.1371/journal.pone.0161059)
Supplement: S3 Table — The test was performed for each pattern category and for the three colonies separately. Decrease or increase of proportions between the two days are indicated with < and >, respectively. Significant P—values (< 0.05) are indicated with *. (DOCX) [file pone.0161059.s005.docx]

| **Colony** | **Category** | **Days** | | | **Proportion day 1** |  | **Proportion day 2** | **X-squared** | **Df** | ***P* - value** |
| --- | --- | --- | --- | --- | --- | --- | --- | --- | --- | --- |
| 1 | dark | 1 | vs | 2 | 0.18 | > | 0.03 | 32.42 | 1 | <0.001* |
| 1 | dark | 2 | vs | 5 | 0.03 | > | 0.00 | 13.45 | 1 | <0.001* |
| 1 | dark | 5 | vs | 8 | 0.00 |  | 0.00 | - | 1 | - |
| 1 | dark | 8 | vs | 12 | 0.00 |  | 0.00 | - | 1 | - |
| 1 | little | 1 | vs | 2 | 0.26 | > | 0.21 | 1.53 | 1 | 0.22 |
| 1 | little | 2 | vs | 5 | 0.21 |  | 0.01 | 94.64 | 1 | <0.001* |
| 1 | little | 5 | vs | 8 | 0.01 | > | 0.005 | 1.73 | 1 | 0.20 |
| 1 | little | 8 | vs | 12 | 0.01 | > | 0.004 | 0.00 | 1 | 1.00 |
| 1 | medium | 1 | vs | 2 | 0.23 | < | 0.40 | 17.00 | 1 | <0.001* |
| 1 | medium | 2 | vs | 5 | 0.40 | > | 0.16 | 57.91 | 1 | <0.001* |
| 1 | medium | 5 | vs | 8 | 0.16 | > | 0.09 | 12.26 | 1 | <0.001* |
| 1 | medium | 8 | vs | 12 | 0.09 | > | 0.04 | 11.25 | 1 | <0.001* |
| 1 | high | 1 | vs | 2 | 0.24 | < | 0.31 | 2.60 | 1 | 0.11 |
| 1 | high | 2 | vs | 5 | 0.31 | < | 0.78 | 168.47 | 1 | <0.001* |
| 1 | high | 5 | vs | 8 | 0.78 | < | 0.84 | 7.19 | 1 | 0.007* |
| 1 | high | 8 | vs | 12 | 0.84 | < | 0.86 | 0.45 | 1 | 0.50 |
| 1 | bright | 1 | vs | 2 | 0.08 | > | 0.05 | 2.31 | 1 | 0.13 |
| 1 | bright | 2 | vs | 5 | 0.05 | < | 0.048 | 0.00 | 1 | 1.00 |
| 1 | bright | 5 | vs | 8 | 0.05 | < | 0.06 | 1.04 | 1 | 0.31 |
| 1 | bright | 8 | vs | 12 | 0.06 | < | 0.10 | 4.75 | 1 | 0.03* |
| 2 | dark | 1 | vs | 2 | 0.47 | > | 0.16 | 38.59 | 1 | <0.001* |
| 2 | dark | 2 | vs | 5 | 0.16 | > | 0.00 | 128.72 | 1 | <0.001* |
| 2 | dark | 5 | vs | 8 | 0.00 |  | 0.00 | - | 1 | - |
| 2 | dark | 8 | vs | 12 | 0.00 |  | 0.00 | - | 1 | - |
| 2 | little | 1 | vs | 2 | 0.20 | < | 0.53 | 34.60 | 1 | <0.001* |
| 2 | little | 2 | vs | 5 | 0.53 | > | 0.01 | 448.93 | 1 | <0.001* |
| 2 | little | 5 | vs | 8 | 0.01 | > | 0.004 | 0.61 | 1 | 0.44 |
| 2 | little | 8 | vs | 12 | 0.004 | > | 0.002 | 0.15 | 1 | 0.70 |
| 2 | medium | 1 | vs | 2 | 0.16 | < | 0.18 | 0.12 | 1 | 0.72 |
| 2 | medium | 2 | vs | 5 | 0.18 | < | 0.55 | 123.59 | 1 | <0.001* |
| 2 | medium | 5 | vs | 8 | 0.55 | > | 0.12 | 303.06 | 1 | <0.001* |
| 2 | medium | 8 | vs | 12 | 0.12 | > | 0.07 | 9.28 | 1 | 0.002* |
| 2 | high | 1 | vs | 2 | 0.12 | > | 0.10 | 0.11 | 1 | 0.74 |
| 2 | high | 2 | vs | 5 | 0.10 | < | 0.43 | 101.19 | 1 | <0.001* |
| 2 | high | 5 | vs | 8 | 0.43 | < | 0.84 | 259.66 | 1 | <0.001* |
| 2 | high | 8 | vs | 12 | 0.84 | > | 0.83 | 0.00 | 1 | 0.98 |
| 2 | bright | 1 | vs | 2 | 0.06 | > | 0.02 | 1.77 | 1 | 0.18 |
| 2 | bright | 2 | vs | 5 | 0.02 | > | 0.01 | 0.83 | 1 | 0.36 |
| 2 | bright | 5 | vs | 8 | 0.01 | < | 0.04 | 10.72 | 1 | 0.001* |
| 2 | bright | 8 | vs | 12 | 0.04 | < | 0.10 | 14.92 | 1 | <0.001* |
| 3 | dark | 1 | vs | 2 | 0.29 | > | 0.12 | 12.04 | 1 | 0.001* |
| 3 | dark | 2 | vs | 5 | 0.12 | > | 0.001 | 90.75 | 1 | <0.001* |
| 3 | dark | 5 | vs | 8 | 0.001 | > | 0.00 | 0.00 | 1 | 1.000 |
| 3 | dark | 8 | vs | 12 | 0.00 |  | 0.00 | - | 1 | - |
| 3 | little | 1 | vs | 2 | 0.27 | < | 0.34 | 1.06 | 1 | 0.30 |
| 3 | little | 2 | vs | 5 | 0.34 | > | 0.001 | 283.28 | 1 | <0.001* |
| 3 | little | 5 | vs | 8 | 0.001 | < | 0.010 | 2.29 | 1 | 0.13 |
| 3 | little | 8 | vs | 12 | 0.01 | > | 0.002 | 1.53 | 1 | 0.22 |
| 3 | medium | 1 | vs | 2 | 0.22 | < | 0.31 | 2.37 | 1 | 0.12 |
| 3 | medium | 2 | vs | 5 | 0.31 | > | 0.22 | 6.92 | 1 | 0.01* |
| 3 | medium | 5 | vs | 8 | 0.22 | > | 0.11 | 26.42 | 1 | <0.001* |
| 3 | medium | 8 | vs | 12 | 0.11 | > | 0.08 | 2.97 | 1 | 0.09 |
| 3 | high | 1 | vs | 2 | 0.17 | < | 0.21 | 0.41 | 1 | 0.52 |
| 3 | high | 2 | vs | 5 | 0.21 | < | 0.77 | 207.55 | 1 | <0.001* |
| 3 | high | 5 | vs | 8 | 0.77 | < | 0.86 | 16.87 | 1 | <0.001* |
| 3 | high | 8 | vs | 12 | 0.86 | < | 0.88 | 1.65 | 1 | 0.20 |
| 3 | bright | 1 | vs | 2 | 0.04 | > | 0.02 | 0.51 | 1 | 0.47 |
| 3 | bright | 2 | vs | 5 | 0.02 | > | 0.01 | 0.29 | 1 | 0.59 |
| 3 | bright | 5 | vs | 8 | 0.01 | < | 0.03 | 1.90 | 1 | 0.17 |
| 3 | bright | 8 | vs | 12 | 0.03 | < | 0.04 | 0.90 | 1 | 0.34 |
