# Supplementary material for: A Look into the Cell: Honey Storage in Honey Bees, Apis mellifera
Source: PLoS One. 2016 Aug 25;11(8):e0161059. doi: 10.1371/journal.pone.0161059 (PMC4999132; doi:10.1371/journal.pone.0161059)
Supplement: S4 Table — Decreasing and increasing values are indicated with < and >, respectively. Significant P-values (< 0.05) are indicated with *. (DOCX) [file pone.0161059.s006.docx]

| **Colony** | **Days** | **Counts** | **Estimate** | **Std. error** | **Z value** | ***P*-value** |
| --- | --- | --- | --- | --- | --- | --- |
| 1 | 1 and 2 | 239 < 277 | 0.15 | 0.09 | 1.67 | 0.10 |
| 1 | 2 and 5 | 277 < 561 | 0.71 | 0.07 | 9.61 | <0.001* |
| 1 | 5 and 8 | 561 < 597 | 0.06 | 0.06 | 1.06 | 0.29 |
| 1 | 8 and 12 | 597 > 579 | -0.03 | 0.06 | -0.53 | 0.60 |
| 2 | 1 and 2 | 109 < 305 | 1.03 | 0.11 | 9.22 | <0.001* |
| 2 | 2 and 5 | 305 < 770 | 0.93 | 0.07 | 13.69 | <0.001* |
| 2 | 5 and 8 | 770 > 690 | -0.11 | 0.05 | -2.09 | 0.04* |
| 2 | 8 and 12 | 690 > 615 | -0.12 | 0.06 | -2.08 | 0.04* |
| 3 | 1 and 2 | 113 < 180 | 0.47 | 0.12 | 3.88 | <0.001* |
| 3 | 2 and 5 | 180 < 819 | 1.52 | 0.08 | 18.41 | <0.001* |
| 3 | 5 and 8 | 819 > 653 | -0.23 | 0.05 | -4.32 | <0.001* |
| 3 | 8 and 12 | 653 < 659 | 0.01 | 0.06 | 0.17 | 0.87 |
