# Supplementary material for: A Look into the Cell: Honey Storage in Honey Bees, Apis mellifera
Source: PLoS One. 2016 Aug 25;11(8):e0161059. doi: 10.1371/journal.pone.0161059 (PMC4999132; doi:10.1371/journal.pone.0161059)
Supplement: S5 Table — (DOCX) [file pone.0161059.s007.docx]

| **Days** | **U** | ***P* - value** |
| --- | --- | --- |
| 0 and 1 | 1 | 0.13 |
| 1 and 2 | 2 | 0.28 |
| 2 and 3 | 2 | 0.28 |
| 3 and 4 | 2 | 0.28 |
| 4 and 5 | 2 | 0.28 |
| 5 and 6 | 5 | 0.83 |
| 6 and 7 | 3 | 0.51 |
| 7 and 8 | 2 | 0.28 |
| 8 and 9 | 3 | 0.51 |
| 9 and 10 | 3 | 0.51 |
| 10 and 11 | 3 | 0.51 |
| 11 and 12 | 3 | 0.51 |
